# Mitochondrial marker implies fishery separate management units for spotted sardinella, *Amblygaster sirm* (Walbaum, 1792) populations in the South China Sea and the Andaman Sea

**DOI:** 10.7717/peerj.13706

**Published:** 2022-07-15

**Authors:** Noorul Azliana Jamaludin, Jamsari Amirul Firdaus Jamaluddin, Masazurah A. Rahim, Noor Adelyna Mohammed Akib, Sahat Ratmuangkhwang, Wahidah Mohd Arshaad, Siti Azizah Mohd Nor

**Affiliations:** 1 Centre for Global Sustainability Studies (CGSS), Universiti Sains Malaysia, Penang, Malaysia; 2 Marine Capture Fisheries Division, Fisheries Research Institute, Sitiawan, Perak, Malaysia; 3 School of Biology, Universiti Sains Malaysia, Penang, Malaysia; 4 Fisheries Research Institute, Penang, Malaysia; 5 Andaman Coastal Research Station for Development, Kasetsart University, Ranong, Thailand; 6 Southeast Asia Fisheries Development Center (SEAFDEC), Marine Fisheries Resources Development and Management Department (MFRDMD), Kuala Terengganu, Terengganu, Malaysia; 7 Institute of Marine Biotechnology, Universiti Malaysia Terengganu, Kuala Terengganu, Terengganu, Malaysia

**Keywords:** Population genetics, Phylogeographic study, Fisheries stock management, Pelagic fish, Sardines

## Abstract

The spotted sardinella, *Amblygaster sirm* (Walbaum, 1792), is a commercial sardine commonly caught in Malaysia. Lack of management of these marine species in Malaysian waters could lead to overfishing and potentially declining fish stock populations. Therefore, sustainable management of this species is of paramount importance to ensure its longevity. As such, molecular information is vital in determining the* A. sirm* population structure and management strategy. In the present study, mitochondrial DNA Cytochrome* b* was sequenced from 10 *A. sirm* populations: the Andaman Sea (AS) (two), South China Sea (SCS) (six), Sulu Sea (SS) (one), and Celebes Sea (CS) (one). Accordingly, the intra-population haplotype diversity (Hd) was high (0.91–1.00), and nucleotide diversity (*π*) was low (0.002–0.009), which suggests a population bottleneck followed by rapid population growth. Based on the phylogenetic trees, minimum spanning network (MSN), population pairwise comparison, and *F*_ST,_and supported by analysis of molecular variance (AMOVA) and spatial analysis of molecular variance (SAMOVA) tests, distinct genetic structures were observed (7.2% to 7.6% genetic divergence) between populations in the SCS and its neighboring waters, versus those in the AS. Furthermore, the results defined *A. sirm* stock boundaries and evolutionary between the west and east coast (which shares the same waters as western Borneo) of Peninsular Malaysia. In addition, genetic homogeneity was revealed throughout the SCS, SS, and CS based on the non-significant *F*_ST_pairwise comparisons. Based on the molecular evidence, separate management strategies may be required for *A. sirm* of the AS and the SCS, including its neighboring waters.

## Introduction

The delineation of marine resources’ population or stock structure is critical for fisheries management and conservation (Shaklee et al., 1999; Maunder & Punt, 2013; Phinchongsakuldit et al., 2013; Prasetyo, Dharmadi & Purwoko, 2019; Ha et al., 2020), particularly for species that are susceptible to commercial exploitation (Garcia et al., 2003; Ha et al., 2020; Ryman & Utter, 1987).

According to Ihssen et al. (1981), a stock is “an intraspecific group of randomly mating individuals with temporal and spatial integrity”. Later, Coyle (1998) redefined it “as a group of fish population inferred to be genetically isolated due to reproductive isolation”. Many biologists consider fish stocks an interbreeding entity originating from a single gene pool. The genetic diversity must be preserved, and population structure patterns considered to optimise resource use (Carvalho & Hauser, 1994). The stock concept equips fishery managers with essential information for sustainable fishery management.

Genetic data is now being incorporated, albeit slowly, in designing marine protected area networks, stock restoration, and fisheries management policies (von der Heyden et al., 2014). These strategies can be formulated once the fishery stock has been genetically defined, to achieve sustainable fishery management. For instance, the common fishery management strategy is suitable for a panmictic population, while non-panmictic fishery stocks warrant different management or policies (Adamson & Hurwood, 2015; Laikre, Palm & Ryman, 2005; Ward, 2000).

Tropical and subtropical sardine species (collectively known as Sardinella), such as herrings, sprats, shads, wolf-herrings, and anchovies from the clupeoid fishes (suborder Clupeoidei and order Clupeiformes) are abundant throughout the Atlantic, Indian, and Pacific oceans (Whitehead, 1985). They are classified into two genera, *Amblygaster* and *Sardinella*, with three species in the former and 22 in the latter. The *Amblygaster sirm* (Walbaum, 1792) or spotted sardinella is one of the small pelagic fishes commonly caught in Southeast Asia. This species is locally known as “Tamban Beluru” in Malaysia (Department of Fisheries Malaysia, 2019) and “Siro” in Indonesia (Suseno et al., 2014). The *A. sirm* is commonly caught using the purse seine gears, a common fish-catching device among Malaysian fishermen (Department of Fisheries Malaysia, 2019). In addition, the species is important in the manufacture of downstream products such as fish crackers and dried salted fish, thus, making it one of the economically important fish in the local seafood enterprise (SEAFDEC, 2017). Recent reports have highlighted the disproportionality in *A. sirm* in harvest production or fish landing numbers in the Southeast Asia waters (SEAFDEC, 2017). This situation needs urgent attention to ensure equitable and continuous production patterns. Nevertheless, there is limited data to develop effective regional strategies for *A. sirm* stock management regulations, especially in this region. Few studies related to stock assessment have been reported on this species with, only a handful conducted in this locality. For instance, stock assessments on reproductive and biological characteristics have been reported in Indian waters (Devi et al., 2018; Pradeep, Shirke & Kar, 2014), a population genetics study in Sri Lanka waters (Aluwihare, Jayasinghe & Dalpathadu, 2019), and a one biomass study in Indonesia (Atmaja & Sadhotomo, 2006).

The series of landing data from 2008 to 2018, indicated an alarming decline of the *A. sirm* population at 30%. In Malaysia, the *A. sirm* stock in the South China Sea decreased from 1,729.95 metric tons (mt) in 2017 to 1,326.58 mt in 2018 (SEAFDEC, 2020a; SEAFDEC, 2020b). The reason underlying this situation remains uncertain. Generally, such declines are associated with overexploitation, the identified cause for the decreasing *Sardinella fimbriata* (SEAFDEC, 2017; SEAFDEC, 2020a; SEAFDEC, 2020b) in Malaysia. Alternatively, a change in management strategies, limited harvest or number of landings resulting from the controlled fishing effort, duration of fishing activities, type of fishing gears, or engine horsepower, gross register tonnage (GRT) (Hale et al., 2015; Wallace & Fletcher, 1996) could also be contribute to this calamity.

Incorporating population genetic data into stock assessment is vital in formulating and implementing a comprehensive management strategy of *A. sirm*. Hypothetically, all the populations are genetically homogeneous or panmictic due to the pelagic nature of the species (Fratini et al., 2016; Garoia et al., 2004; Hauser & Ward, 1998). Malaysia and its neighboring countries should develop a common sustainable fisheries management strategy if the results support this hypothesis. On the other hand, separate management for each evolutionary unit is required if the hypothesis is rejected. The importance of population genetics data has been acknowledged for the fishery management of Spanish mackerel, *Scomberomorus commerson* in the northern Indian Ocean (Radhakrishnan et al., 2018); tiger shark, *Galeocerdo cuvier* across the Indo-Pacific Ocean (Holmes et al., 2017); Chinese loaches, *Misgurnus mohoity* and *M. bipartitus* in Northeast China (Yi et al., 2016) and Asian green mussel, *Perna viridis* along the Indian coast (Divya et al., 2020).

In the present study, the mitochondrial DNA (mtDNA) *Cytochrome b* (*Cyt b*) gene was utilized to elucidate the population genetic structure of spotted sardinella, *A. sirm,* in Malaysian waters and to establish a management plan for this species. This protein-coding gene has been proven to be highly efficient in determining intra and inter-specific and higher-level phylogenetic relationships (Roques et al., 2006; Schönhuth & Mayden, 2010; Zhang et al., 2018). The mtDNA *Cyt b* gene is also one of the most extensively sequenced genes to date, and the evolutionary dynamics have been intensively characterised. Furthermore, this gene has been widely applied in systematic studies to investigate divergence at taxonomic levels (Peng, He & Zhang, 2004; Habib et al., 2011; Rahim et al., 2012; Schmidt, Bart & Nyingi, 2018).

The study outcomes are essential for fishery managers to develop sound fishery strategies across regions and countries to prevent the irreversible decline of resources. Population stock study is essential to support resource recovery through improved knowledge of stock delineation. This knowledge will pave the way forward effective monitoring of populations, as suggested by previous studies on other pelagic species such as longtail tuna, *Thunnus tonggol* (Kasim et al., 2020). Additionally, this will guarantee food security and decrease the poverty level in the community, which is of high priority to the nation, and to achieve sustainable development goals (SDG). The SDG was introduced by the United Nations in 2015 and comprises 17 goals (starting from SDG 1 to SDG 17) designed for a more sustainable future. The specific target for this study is SDG14 (life below water). In addition, the output from this study would be valuable for the conservation and management of *A. sirm* in Malaysian waters.

## Materials and Methods

### Ethical statement

Since only dead specimens were sampled, no permit was required, and no ethical consideration were required for this study. In addition, *A. sirm* is not listed under the International Union for Conservation of Nature (IUCN) list of endangered or protected species.

### Sample collection

**Figure 1:**
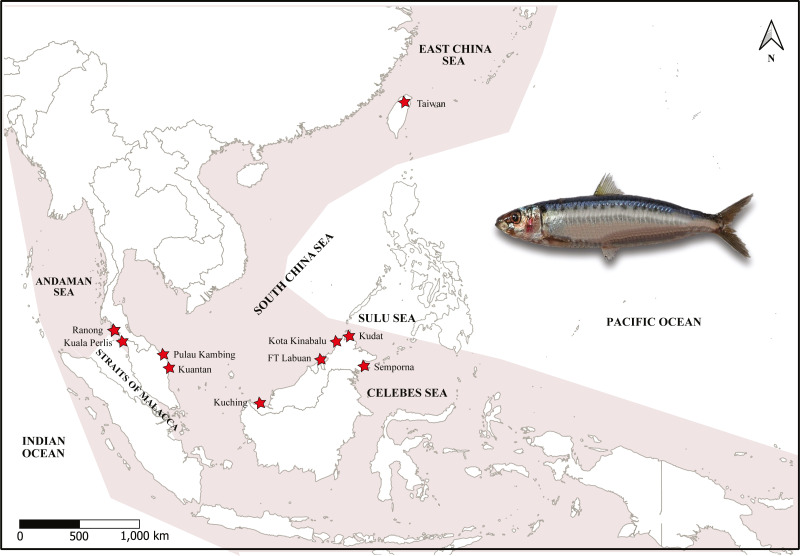
Map of sampling sites, targeted species and haplotype networks. Map of sampling sites and specimen of *A. sirm* (Walbaum, 1792). The shaded areas indicate the distribution according to Whitehead (1985). The collection localities are indicated with red stars.

A total of 179 specimens of *A. sirm* were collected from ten landing sites from vessels that used purse seine fishing gears in Peninsular Malaysia, Sabah, and Sarawak from 2015 to 2019. These sampling locations cover the southern region of the SCS, SS, CS, and AS. The species were morphologically identified by of 10 to 20 series of black spots down the flank, according to Whitehead (1985) (Table 1; Fig. 1).

**Table 1:** Sampling locations, coordinates, collection date, sample size, historical demographic analyses and population genetic statistics of 10 *A. sirm* populations based on mtDNA Cyt *b* region sequences.

**Sampling locations**	**Sea**	**Sampling date**	**Latitude/Longitude**	**N**	***h***	**PS**	**Hd**	*π*	**Tau**	***Hri***	**Tajima’s D**	**Fu’s F**	*θ* **0**	*θ* **1**	**R**_**2**_
**Ranong (SRG)**	AS	15.12.2019	9°55′52″N 98°24′30″E	11	9	22	0.96	0.006	7.5	0.10	−0.59^*^	−1.98^*^	0.06	29.33	0.121
**Kuala Perlis (SKP)**		6.12.2018	6°24′1” N 100°7′51″E	10	8	36	0.97	0.009	1.5	0.04	−1.25^*^	−1.77^*^	10.63	3589.03	0.133
**Pulau Kambing (SPK)**	SCS	15.1.2018	5°21′14″N 103°8′35″E	16	13	27	0.95	0.005	4.4	0.05	−1.54	−4.56	3414.98	74.68	0.057
**Kuantan (SKT)**		22.1.2018	3°45′32″N 103°23′10″E	35	24	30	0.95	0.003	2.9	0.04	−2.19	−23.20	0.00	1.14	0.034
**Kuching (SKC)**		12.3.2015	1°44′10″N 110°38′28″E	34	25	39	0.96	0.003	3.9	0.02	−2.15	−20.40	0.04	58.92	0.034
**Labuan (SLB)**		23.10.2019	4°32′42″N 118°36′14″E	12	10	14	0.97	0.004	4.1	0.05	−0.95^*^	−5.00	0.00	33.55	0.042
**Kota Kinabalu (SKK)**		6.1.2018	5°59′13″N 116°1′58″E	15	15	46	1.00	0.008	4.0	0.03	−1.73	−8.42	5.50	3415.00	0.085
**Taiwan (STN)**		8.2.2018	22°53′24″N 122°20′59″E	2	2	2	–	–	–	–	–	–	–	–	–
**Kudat (SKD)**	SS	11.5.2015	6°53′13″N 116°49′30″E	35	21	24	0.91	0.002	2.0	0.05	−2.08	−18.85	0.45	59.06	0.039
**Semporna (SSP)**	CS	6.1.2018	5°22′8″N 115°11′2″E	9	9	21	1	0.005	4.0	0.04	−1.26^*^	−4.40	1.9	3415.00	0.093
**TOTAL**				179					**3.8**	**0.046^a^**	**−0.152^a^**	**−9.84^a^**	**2.255**	**2775.26**	**0.077**

**Notes.**

SCS South China Sea AS Andaman Sea SS Sulu Sea CS Celebes Sea N number of individuals *h* number of haplotypes PS polymorphic sites Hd Haplotype diversity *π* nucleotide diversity *Hri* Harpending’s raggedness index *θ*_0_/ *θ*_1_ before/after expansion R _2_ Ramos- Onsins and Rozas

* Significant value after False Discovery Rate Procedure (FDR) procedure at *p* <0.05.

Totals are indicated in bold.

In addition, a small clipping of *A. sirm* pectoral fin was collected during the sampling activity. Each clipping was fixed in a separate vial containing 95% ethanol and stored at −20 °C until further use.

The sampling locations were divided into four regions following Kasim et al. (2020). The details of each landing port were obtained via interviews with fishermens/boatmen and confirmed by consulting with the Department of Fisheries staff stationed at the landing sites. Notably, no samples were obtained from the Strait of Malacca (a waterway that links the AS and the SCS), as *A. sirm* is absent from this location (Whitehead, 1985). Meanwhile, sample collections in Ranong, Thailand, were facilitated by the Andaman Coastal Research Station for Development, Kasetsart University of Thailand. Two samples from Taiwan were contributed by collaborators from the National University of Taiwan and the National Museum of Marine Biology, Taiwan (see Fig. 1). Due to the low number of samples (*N* = 2), the data was only used in the phylogenetic analysis.

### Extraction and polymerase chain reaction (PCR) amplification

Genomic DNA extraction of 179 samples was performed using the DNeasy Blood & Tissue Kit (Qiagen, Hilden, Germany) according to the manufacturer’s protocol. The quality of extracted DNA was assessed using UV spectrophotometer Q3000 (Quawell, San Jose, CA) and diluted to a final concentration of 50 ng/µL. The mtDNA *Cyt b* was targeted using the following primers; F: WMA15-F (5′ACC GTT GTA ATT CAA CTA TAG AAA C 3′) and R: TruCytb-R (5′ CCG ACT TCC GGA TTA CAA GAC CG 3′) (Jérôme et al., 2003). The PCR amplification was conducted in a Thermal Cycler (Techgene, Irving, TX, USA) at a final volume of 25 µL, composed of 12.5 µL of 2x EasyTaq^®^ SuperMix (TransGen Biotech, Beijing, China), 0.2 µL of each primer, 2.0 µL of genomic DNA (50 ng/mL), followed by nuclease-free water to achieve the final reaction volume. The thermal cycling conditions were as follows: initial denaturation at 95 °C for 2 min, followed by 35 cycles of denaturation at 94 °C for 10 s, annealing at 56.8 °C for 10 s, and extension at 72 °C for 15 s. The quality of PCR products was visualised on 1.5% agarose gels stained with 1 to 3 µL of GelRedTM Nucleic Acid Gel Stain (Biotium Inc., Fremont, CA, USA). The unpurified PCR amplicons were sent to Repfon Technologies Sdn Bhd for purification and sequencing in forward and reverse directions in an automated sequencer (ABI3730xl, Applied Biosystems, Bedford, MA, USA).

### Data analysis

The raw sequences for both forward and reverse sequences were edited and assembled after removing the primer sites.The final assembled sequences were aligned with ClustalW implemented in MEGA v7.0 with default setting (Kumar et al., 2018). The complete aligned data set was inspected for nucleotide variable sites, parsimony informative sites, number of haplotypes, haplotype distributions, transitions and transversions, and nucleotide frequencies in DnaSP v6.0 (Rozas et al., 2017). A simple linear regression analysis (Pearson correlation test) was conducted to assess whether sample size (N) would affect the downstream analyses to ensure the statistical validity of further investigations. All the statistical analysis were performed using the IBM SPSS Statistics for Windows software, version 23.0 (IBM Corp. Armonk, NY, USA). Since the initial analysis demonstrated that the sample sizes did not affect the haplotype diversity (Hd) and nucleotide diversity (*π*) (*p* > 0.05) therefore, further analysis could be proceeded (Castillo-Páez et al., 2014; Delrieu-Trottin, Maynard & Planes, 2014).

### Phylogenetic and genetic diversity analyses

The RAxML (Stamatakis, Hoover & Rougemont, 2008) and maximum likelihood (ML) trees, with 1,000 bootstrap replicates, were constructed in raxmlGUI v1.5b1 (Silvestro & Michalak, 2012) using a general time-reversible model of nucleotide substitution with a heterogeneity rate following a discrete gamma distribution (GTR+G) as the default selection. Bayesian inference (BI) was carried out in MrBayes v.3.2 (Ronquist & Huelsenbeck, 2003) with 1,000,000 Markov Chain Monte Carlo (MCMC) run generations; posterior distribution was sampled every 1,000 generations, and a 25% *burn-in.* GenBank sequences of *S. hualensis* (Acc No: KC951523), *S. lemuru* (NC039553), and *S. gibbosa* (NC037131) were employed as outgroups in the analysis for both trees. A phylogenetic network of all haplotypes was constructed to view haplotype relationships based on the median-joining calculation in a minimum spanning network (MSN) implemented in the population analysis with reticulate trees (POPART) v1.7 (Bandelt, Forster & Röhl, 1999; Leigh & Bryant, 2015).

Genetic distances within and among populations based on the best nucleotide substitution model of Kimura 2P (K2P), which depicts the lowest Bayesian information criterion (BIC) score, were estimated using MEGA v7.0. These values also assessed the possibility of sub-species or cryptic species occurrence if intra-species variation within marine species exceeds the threshold (2%) (Chanthran et al., 2020; Hebert et al., 2003; Jamaludin et al., 2020; Ward, 2009).

### Population structure

Population pairwise comparisons (*F*_*ST*_) were calculated using ARLEQUIN v3.5 (Excoffier & Lischer, 2010). Values were adjusted for Type 1 errors as a result of multiple comparisons using the false discovery rate procedure (FDR) at *p* < 0.05 (Benjamini & Hochberg, 1995). The genetic distance and significance of each pairwise comparison were further analysed with a nonparametric permutation procedure with 1,000 replicates (Hudson, Boos & Kaplan, 1992) in DnaSP v6.0 (Rozas et al., 2017). Estimation of gene flow (Nm) based on both haplotype and sequence statistics was derived according to Nei (1973) and Hudson, Boos & Kaplan (1992) using the same programme.

Hierarchical analysis of molecular variance (AMOVA) was performed to estimate molecular variance among populations at different hierarchical levels using ARLEQUIN v3.5 (Excoffier & Lischer, 2010). The relative contribution of variances was estimated at three different levels; *F*_*ST*_, *F*_*SC*_, and *F*_*CT*_ (Excoffier & Lischer, 2010). The spatial structure was further examined using spatial analysis of molecular variance (SAMOVA) v2.0 (Dupanloup, Schneider & Excoffier, 2002) to identify groups of populations that were geographically homogeneous and maximally differentiated from each other. The isolation by distance (IBD) or Mantel test (Mantel, 1967) was conducted to confirm the relationship between genetic distance and geographical distance.

### Demographic history

The historical demography of each population was tested using Tajima’s D (Tajima, 1989) and Fu’s F (Fu, 1997) statistics in ARLEQUIN v3.5 (Excoffier & Lischer, 2010) Ramos-Onsins and Rozas’ R_2_ in DnaSP v6.0 (Rozas et al., 2017) to analyse deviation from neutrality. The significant R_2_ was calculated using coalescent simulations of 5,000 replicate runs for each simulation. R_2_ is a powerful tool for quantifying population growth with a limited sample size (Ramos-Onsins & Rozas, 2002). Harpending’s raggedness index, H*ri* (Harpending, 1994), was calculated in ARLEQUIN v3.5 (Excoffier & Lischer, 2010) and mismatch distributions (Rogers & Harpending, 1992; Schneider & Excoffier, 1999; Slatkin & Hudson, 1991) were calculated in DnaSP v6.0 (Rozas et al., 2017). Both analyses could differentiate whether populations are demographically stable, expanding, or decreasing over time. A sudden population expansion model in H*ri* will be rejected if *p* < 0.05 (Schneider & Excoffier, 1999).

The precise time of expansion was manually calculated using the equation *τ* = 2 µt, (µ= mutation rate of the sequence analysed, t = time since expansion) with a given mutation rate of 1–2% per million years for *Cyt b* (Rousset, 1997; Johns & Avise, 1998; Cárdenas et al., 2005). The Bayesian skyline plot (Drummond et al., 2012) was implemented in BEAST v1.8.2 (Drummond et al., 2012) to estimate past population dynamics, employing a relaxed uncorrelated lognormal molecular clock, GTR + G as the best evolutionary model selected from PartitionFinder v1.1.0 (Lanfear et al., 2017). The analysis was run for 300 million generations with parameters sampled every 10,000 generations. The results were visualised using Tracer v1.7 (Rambaut et al., 2018) which summaries the posterior distribution of population size over time.

## Results

### Data analysis

The 1,016 bp segment of mtDNA *Cyt b* was successfully amplified from all 179 samples. A detailed evaluation revealed 42 non-synonymous mutations resulting in 37 amino acid substitutions. The ratio of transversion to transition substitutions for the entire data set was 1:1. Haplotype diversity (Hd) was high ranging from 0.91 to 1.00, while nucleotide diversity (*π*) was low, ranging from 0.002 to 0.009 (see Table 1). A total of 203 variable sites were identified from the 1,016 bp segment, where 111 (10.9%) parsimony informative sites defined 122 haplotypes. These haplotype sequences have been deposited in GenBank under accession numbers MZ040756 to MZ040877. Furthermore, 111/122 (90.98%) were detected as singletons (haplotypes found in a single gene copy), where 11/122 (9.02%) were shared haplotypes or found in more than a single population. The Pearson correlation test (r^2^ = 0.66, *p* = 0.06) confirmed that sample sizes did not significantly affect the nucleotide diversity and haplotype diversity (Hd) therefore, further analyses could be performed.

### Phylogenetic relationships

**Figure 2:**
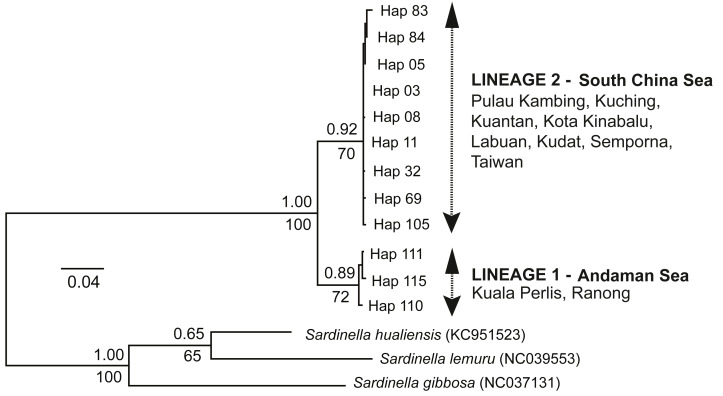
Maximum likelihood (ML) tree and Bayesian inference (BI). The ML tree and BI analysis from the partial mtDNA Cyt *b* gene of *A. sirm* (the singletons and population-specific haplotypes are excluded in the tree for better illustration).

The ML tree and BI analysis revealed a similar topology of two geographically separate mtDNA lineages (see Fig. 2); (1) AS lineage (Lineage 1) composed of two populations (Ranong and Kuala Perlis), and (2) SCS lineage (Lineage 2) encompasses populations from the SCS, CS, and the SS. No geographical pattern was observed within Lineage 2.

**Figure 3:**
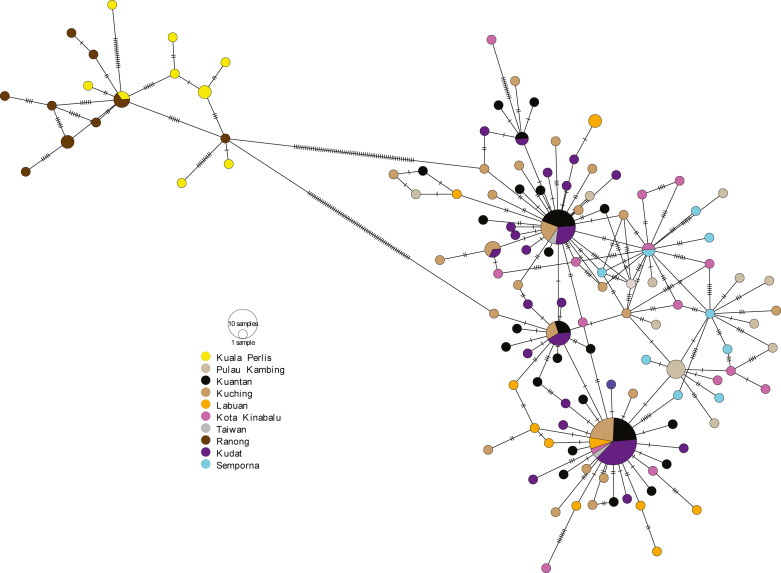
Minimum spanning network (MSN). The MSN inferred from mtDNA Cyt *b* gene of *A. sirm.* The node size corresponds to haplotype frequencies; the minimum node size is one individual. The dashed line represents a genetic mutation.

The minimum spanning network (MSN) showed two distinct lineages similar to the phylogenetic trees, separated by 63 genetic mutations (see Fig. 3). Lineage 2 (all populations excluding AS populations) exhibited a star-like pattern with the dominant haplotype (Hap05) centred in it (Ferreri, Qu & Han, 2011; Woolley, Posada & Crandall, 2008).

### Genetic diversity

The genetic diversity or distance was converted to percentages and revealed intrapopulation genetic diversity ranging from 0.2% to 0.9%, the lowest being in Kudat and the highest in Kuala Perlis (Table 2). Meanwhile, the interpopulation genetic diversity ranged between 0.2% and 7.6%, with Kuala Perlis and Ranong from the AS being the most divergent of all (7.2% to 7.6%), with a 1.0% difference from each other. In contrast, the genetic diversity ranged from 0.2% to 0.7%, when Kuala Perlis and Ranong populations were excluded from the analysis, providing strong evidence that AS populations are highly structured than other seas.

**Table 2:** Population pairwise, *F*_*ST*_ (below diagonal) and genetic diversity (distance) (upper diagonal) of *A. sirm* inferred by mtDNA *Cyt b*.

		**Andaman Sea**		**South China Sea**					**Celebes Sea**	**Sulu Sea**	
		**SKP**	**SRG**	**SKC**	**SKT**	**SPK**	**SKK**	**SLB**	**SKD**	**SSP**	
		0.009	0.001	0.073	0.073	0.075	0.076	0.073	0.073	0.076	**SKP**
			0.007	0.072	0.072	0.074	0.076	0.072	0.072	0.075	**SRG**
**Andaman Sea**	**SKP**			0.003	0.005	0.005	0.006	0.004	0.003	0.005	**SKC**
	**SRG**	0.0114			0.003	0.003	0.006	0.003	0.002	0.005	**SKT**
**South China Sea**	**SKC**	**0.0293**	**0.0358**			0.005	0.007	0.005	0.005	0.005	**SPK**
	**SKT**	**0.0600**	**0.0662**	−0.0038			0.008	0.006	0.006	0.007	**SKK**
	**SPK**	**0.0386**	**0.0450**	**0.0456**	0.0545			0.004	0.003	0.006	**SLB**
	**SKK**	**0.0404**	**0.0471**	**0.0165**	**0.0273**	0.0286			0.002	0.005	**SKD**
	**SLB**	**0.0107**	**0.0177**	−0.0004	0.0088	**0.0444**	**0.0148**			0.006	**SSP**
**Celebes Sea**	**SKD**	**0.0263**	**0.0333**	−0.0024	−0.0031	**0.0751**	**0.0480**	0.0080			
**Sulu Sea**	**SSP**	0.0112	0.0186	**0.0193**	**0.0287**	0.0301	−0.0075	0.0156	**0.0504**		

**Notes.**

SKP Kuala Perlis SRG Ranong SKC Kuching SKT Kuantan SPK Pulau Kambing SLB FT Labuan SKD Kudat SSP Semporna

Bold number indicates significant value after False Discovery Rate Procedure (FDR) procedure at *p* < 0.05.

The genetic diversity (distance) values were presented in percentage (%) in the text.

### Population structure

Most *F*_*ST*_ values in the AS (Kuala Perlis and Ranong) populations were significantly different (*p* < 0.05) (Table 2), which aligned with earlier studies. However, non-significant (*p* > 0.05) values were also detected in cases where high genetic distance in some between Lineage 1 and Lineage 2 populations, such as AS *vs* Semporna Kuantan *vs* Kuching (both in SCS), Labuan (SCS) *vs* Kudat (SS), and Semporna (CS) *vs* Kuantan (SCS).

The AMOVA indicated no significant differences among populations (between seas) even for the highest variance (*F*_*CT*_ = 0.0066, *p* = 0.257; Table 3). However, the dataset demonstrated significant differences within each sea (*F*_*ST*_ = 0.0265, *p* = 0.0009), with 97.34% of the total genetic variation of *A. sirm* contributed by genetic differences among the total populations. This result indicated a subdivision or structuring within the tested populations. Notably, populations may be contribute the non-significant (*p* > 0.05) observation within the SCS, represented by more than two populations compared to other seas. Genetic differentiation among populations within groups/seas was significant (*F*_*SC*_ = 0.0202, *p* = 0.03).

**Table 3:** Hierarchical AMOVA analysis of *A. sirm* inferred by Cyt *b* based on nine populations.

**Source of variation**	**Variation (%)**	**F statistics**	***p*** **value**
Among populations	0.66	*F*_*CT*_ = − 0.0066	0.257
Among populations within groups	2.00	*F*_*SC*_ = 0.0202	0.003
Among populations within total	97.34	*F*_*ST*_ = 0.0265	0.0009

**Notes.**

The populations were grouped into seas:

Group 1-AS Kuala Perlis (SKP) and Ranong (SRG) Group 2-SCS Kuching (SKC), Kuantan (SKT), Kota Kinabalu (SKK), Pulau Kambing (SPK) and Labuan (SLB) Group 3-SS Kudat (SKD) Group 4- CS Semporna (SSP)

The result from the SAMOVA paralleled other analyses, with *k* = 2 displaying the highest *F*_*CT*_ value (*F*_*CT*_ = 0.9277, *p* = 0.02) signifying two genetically distinct *A. sirm* stocks among sampled populations: Lineage 1 (Kuala Perlis and Ranong) and Lineage 2 (Kuching, Kota Kinabalu, Kuantan, Labuan, Pulau Kambing, Kudat and Semporna) (Supplemental Information 1). The Mantel test showed no significant correlation between genetic differentiation (*F*_*ST*_ value) and geographical distance (*r* = 0.127, *p* = 0.173) among tested populations.

### Demographic history

**Figure 4:**
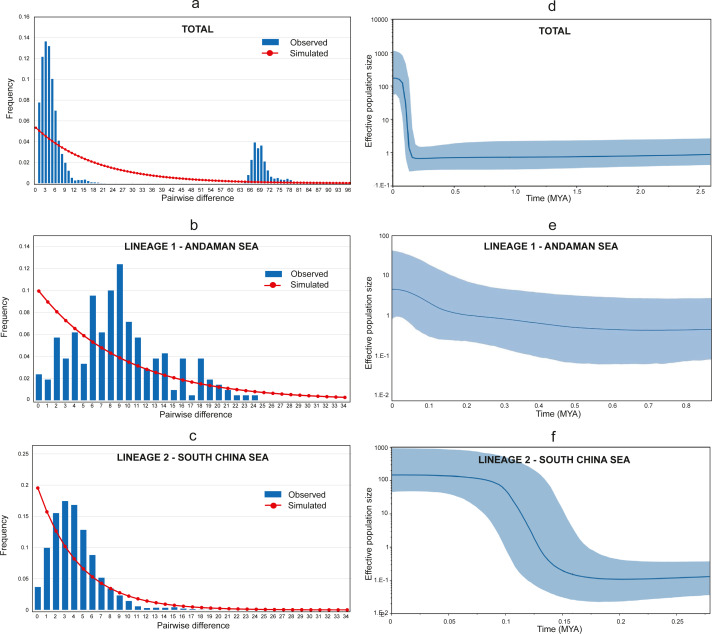
Mismatch distribution and Bayesian Skyline Plot (BSP). (A) Mismatch distribution (pairwise number of differences) for AS and SCS. (B) Mismatch distribution (pairwise number of differences) for AS lineage (Lineage 1). (C) Mismatch distribution (pairwise number of differences) for SCS lineage (Lineage 2). (D) The BSP (right) of the effective population size for mtDNA Cyt *b* for *A. sirm* combining the nine populations. (E) The BSP (right) for AS lineage (Lineage 1). (F) The BSP (right) for SCS lineage (Lineage 2). The shaded area indicates 95% confidence intervals surrounding the median.

The Tajima D’s and Fu’s F generated negative and significant (*p* < 0.05) values for all populations (Table 1) suggesting a recent historical directional selection (selective sweep) or recent population growth (Tajima, 1989). Furthermore, the Harpending raggedness index, *Hri* = 0.042, SSD = 0.01, (*p* = 0.3) supported the recent population growth hypothesis. Meanwhile, the mismatch distributions of combined populations detected two highly divergent peaks, representing the two lineages (see Fig. 4A). Additional independent analyses of the two lineages (Lineage 1 and Lineage 2) were conducted, and a bimodal mismatch distribution was discovered in Lineage 1 (Fig. 4B). Nevertheless, further comprehensive studies are required to confirm these preliminary finding due to the small sample size in this study. On the other hand, Lineage 2 shows a unimodal distribution providing strong evidence for sudden population expansion (Harpending, 1994; Slatkin & Hudson, 1991) (see Fig. 4C).

The Bayesian skyline plot analyses revealed a recent expansion in the overall effective population size (see Fig. 4D), which can be explained by growth in Lineage 2 occurred approximately around 150 thousand years ago (KYA) (see Fig. 4F). Conversely, Lineage 1 showed a gradual population increase over the last 500 KYA (see Fig. 4E). Notwithstanding, the finding was based on only two populations from the same sea and may not represent other similar sites. The estimated time of population expansion for Lineage 1 and Lineage 2 using the formula *τ* = 2 *μ*t, generations and a mutation rate for Cyt *b* of 1–2% per million years for perciform (Cárdenas et al., 2005; Johns & Avise, 1998) are estimated to occur 442,913 to 221,456 years ago and 354,330 to 177,165 years ago respectively. These findings are almost in parallel with the results from the Bayesian Skyline plot suggesting a demographic expansion in the *A. sirm* population during the Pleistocene era.

## Discussion

In the absence of any form of barriers, genetic homogeneity or absence of spatial patterns in allele or haplotype distributions is expected in the marine fish population (Baker et al., 1993; Scoles & Graves, 1993; Akib et al., 2015). However, this theory become obsolete when barriers impede the free movement of a particular species (Tudela, García-Marín & Pla, 1999; Zardoya et al., 2004; Gaggiotti et al., 2009; Sukumaran, Sebastian & Gopalakrishnan, 2017; Swart et al., 2016, Domingues et al., 2018; Jamaludin et al., 2020). In this study, two genetically different groups, Lineage 1 (AS populations) and Lineage 2 (SCS and the neighboring waters populations) were identified based on various statistical analyses. However, the exact position of this boundary was not identified since no sample was obtained from the Strait of Malacca that connects the Indian Ocean and the SCS. Several studies have reported the phenomena in broad spectrum of marine organisms (Ninwichian & Klinbunga, 2020; Klangnurak, Phinchongsakuldit & True, 2012; Mandal et al., 2012) in the AS or even in the SCS (Rohfritsch & Borsa, 2005; Dudgeon, Broderick & Ovenden, 2009; Dohna et al., 2015; Swart et al., 2016; Jamaludin et al., 2020). Examples of other marine organisms include *Tenualosa macrura* (Longtail shad), *Scarus ghonnan* (Blue-barred parrotfish), and *Sepioteuths lessoniana* (Bigfin reef squid) (Department of Fisheries Malaysia, 2019), although they are common in the surrounding seas. Further investigation is warranted to determine the underlying factor(s) if this species is absent from the area.

In addition to the two disparate lineages, significant divergences (*F*_*ST*_) were also observed for several pairwise comparisons within Lineage 2 involving Kuching, Kudat, Kuantan, Kota Kinabalu, and Pulau Kambing. In contrast, non-significance was observed between Lineage 1 and Semporna (Lineage 2). Upon weighing other evidence, such as the phylogenetic tree and MSN, it was predicted that the statistical sensitivity of *F*_*ST*_ was contributed by the small sample sizes rather than genetic isolation or other biological factors.

Many studies have refuted the universality of free gene flow in marine species (Zhang et al., 2016; Li et al., 2016; Niu et al., 2019; Akbar, Irfan & Aris, 2019; Kasim et al., 2020; Mat Jaafar et al., 2020). Alternatively, several general factors have been proposed to explain the population structure pattern of marine fish, including random genetic drift, demographic history, and life histories such as feeding and migratory behaviour (Gaggiotti et al., 2009; Sun, Tang & Yin, 2018). In addition the limited dispersal and geomorphologic barriers could be possible reasons for the restricted gene flow in *A. sirm,* as observed in other species (Betancur et al., 2010). Dispersal ability and feeding behaviour are hypothesised to be driven by oceanic currents that could lead to allopatric speciation, as documented in the Mapalé sea catfish, *Cathorops mapale* in Southern Caribbean waters (Betancur et al., 2010). Thus, extrinsic factors (circulation patterns) and intrinsic factors (limited dispersal ability) are potent drivers of genetic differentiation.

In the absence of (historical and contemporary factors) movement barriers, the adult and larval pelagic movement characteristics are sufficient for homogenising populations, as observed in the panmictic *A. sirm* populations within the SCS and neighboring seas (Lineage 2). The non-significant *F*_*ST*_ values among Lineage 2 populations indicated low genetic divergence and high gene flow. This condition results from the active exchange of genetic material between populations through unrestricted breeding, concordant with this migratory species’ pelagic migratory behavior. High genetic affinity was also observed between the AS populations in Kuala Perlis and Ranong. Similar findings have been recorded in other pelagic fishes in the region, such as ornate threadfin bream, *Nemipterus hexodon* (Supmee et al., 2021); crescent perch, *Terapon jarbua* (Chanthran et al., 2020), Japanese scad, *Decapterus maruadsi* (Jamaludin et al., 2020), Russell’s snapper, *Lutjanus russelli* (Klangnurak, Phinchongsakuldit & True, 2012); lesser spotted-leatherjacket, *Thamnaconus hypargyreus* (Li et al., 2016) and crimson snapper*, Lutjanus erythropterus* (Zhang, Cai & Huang, 2006). Furthermore, the drifting planktonic larva could further enhance the genetic connectivity of *A. sirm* in SCS, a factor attributed to the panmixia observed in the ornate threadfin bream, *Nemipterus hexodon* along the Gulf of Thailand coastline (Supmee et al., 2021). Larval transport due to oceanic water movement during monsoons is suggested to strongly influence the population’s geographical structure (Palumbi, 1994). Nonetheless, this hypothesis warrants future investigations for the *A. sirm* population.

Climatic history may be pivotal in shaping the demographic history of *A. sirm.* Hou & Li (2018) suggested that a break in the Tethyan ocean due to geological changes affected the aquatic diversification, including in the Indo-West Pacific region. The demographic history of high haplotype diversity, Hd coupled with low nucleotide diversity *π* for *A. sirm* propose a recent population expansion from a low effective size, a probable consequence of rapid population growth after a bottleneck event resulting in a high occurrence of new mutations (Avise, Neigel & Arnold, 1984). This finding aligned with previous studies on pelagic species in this region; Indian mackerel, *Rastrelliger kanagurta* (Akib et al., 2015); Japanese scad, *Decapterus maruadsi* (Jamaludin et al., 2020), yellowfin tuna, *Thunnus albacares*; skipjack, *Katsuwonus pelamis* (Ely et al., 2005) and spotted mackerel, *Scomber australasicus* (Tzeng, 2007).

The ocean level fluctuations during the Pleistocene significantly impacted the dispersal of marine and global species (Voris, 2000), including *A. sirm*. Morevoer, this occurence could have reshaped the geographical landscape of the AS. When ocean levels increased, populations within the SCS and the neighboring waters were homogenised. This genetic structuring pattern has been highlighted in the Japanese scad, *Decapterus maruadsi* (Jamaludin et al., 2020), redlip mullet, *Chelon haematocheilus* (Liu et al., 2007) and crimson snapper, *Lutjanus erythropterus*, (Zhang, Cai & Huang, 2006) and various other marine species (Carpenter et al., 2011). When the sea level contracted, mainland Southeast Asia was exposed during the Quaternary period (Nelson et al., 2000), leading to the high genetic divergence between the AS and the SCS. Chanthran et al. (2020) attributed the genetic separation between the Kuala Selangor (Peninsular Malaysia) and Sandakan (Borneo) populations of crescent perch, *Terapon jarbua* to this factor*.* The same factor underlies the separation of the ornate threadfin bream, *Nemipterus hexodon* (Supmee et al., 2021), and Russell’s snapper (Klangnurak, Phinchongsakuldit & True, 2012) between the AS and the Gulf of Thailand. Similarly, *A. sirm* may experience this phenomenon due to the high genetic distance between the SCS and the AS.

The small sample size (in Semporna) and number of populations (only two populations in the AS *vs*eight in the SCS and the neighboring waters) in the present may have influenced the statistical strength of the observed population structuring. Nevertheless, MSN, AMOVA and SAMOVA analysis validated this hypothesis. Furthermore, there was no haplotype sharing between the two lineages. Future studies should consider larger sample sizes and populations from the AS to verify the present findings since this research had limited geographical coverage. Furthermore, sampling could be extended to include other areas, such as the Bay of Bengal, where close genetic relatedness with the AS had been identified in several species. This observation was exemplified in the widely distributed Central Indo-Pacific surgeonfishes, *Naso brevirostris* and *N. unicornis* (Horne et al., 2008), Indian mackerel, *Rastrelliger kanagurta* in Indian Peninsular waters (Sukumaran, Sebastian & Gopalakrishnan, 2017) and Spanish mackerel, *Scomberomorus commerson* in the Northern Indian ocean (Radhakrishnan et al., 2018). The high genetic affinity of these species populations was documented in the Indian Ocean—the AS and Bay of Bengal are part of this vast ocean. The was also evident in other *Sardinella* spp. in other regions. For instance, the low genetic differentiation of white sardine, *S. albella,* between the Persian Gulf and Sea of Oman was attributed to the free dispersal of currents in the Indian Ocean seas (Rahimi et al., 2016). On the other hand, Bowen & Grant (1997) observed that the *Sardinops* spp. experienced a long and stable evolutionary history and has a wide phylogeographic distribution in temperate upwelling zones in the coastal region of the Indian Ocean and Pacific Oceans, such as Japan, California, Chile, Australia, and South Africa. Therefore, the inclusion of *A. sirm* populations from the Bay of Bengal area (India and Sri Lanka) in future studies should be explored. However, the current findings are based solely on a maternally inherited gene. Thus, co-dominant markers such as microsatellites, single nucleotide polymorphisms (SNPs), or whole genome sequencing should be integrated into future studies.

The present of cryptic species or sub-species due to high genetic differentiation is not uncommon among *Sardinella* species, indicated by the high genetic differentiation between the two lineages in this study. The genetic distance between Lineage 1 and Lineage 2 (∼7%) exceeded the threshold (2%) of intra-species variation within marine species (Chanthran et al., 2020; Hebert et al., 2003; Jamaludin et al., 2020; Ward, 2009). Furthermore, high genetic differentiation in the waters of the Philippines was attributed to the cryptic diversity of freshwater sardinella, *S. tawillis* (>3%) Taiwan sardinella, *S. hualensis* (>4%) (Chan et al., 2019) and the Goldstripe sardinella, *S. gibbosa* (no specific genetic percentage was reported but the genetic differences were supported by morphological examination) (Thomas et al., 2014). Nonetheless, cryptic diversity can only be verified through a more comprehensive and holistic approach utilising complementary tools (Jamaludin et al., 2020; Jayasankar et al., 2004; Thomas et al., 2014) and morphological re-examining of fresh type specimens.

### Implications to fishery management

This study has provided novel insights into the population structure and proposed several processes that could define the stock boundaries and evolutionary units of *A. sirm* in waters. It is recommended that separate management systems be implemented for the waters fringing the western coasts, Lineage 1 (AS) and eastern coasts, Lineage 2 (the SCS and the neighboring waters). Despite focusing on only one country, it is worth notingy that all the major seas in Southeast Asia are represented in this study.

Genetic data should be an important component for fishery managers to make informed decisions on a target species (von der Heyden et al., 2014). Furthermore, advanced genetic techniques (eDNA for ecological monitoring) could provide crucial information to the scientific advisory process for fisheries management. Despite that, the integration of genetic data into fishery policy modeling is yet to gain traction in most parts of the world where genetic facilities are still lacking (Reiss et al., 2009; Waples & Naish, 2009), including Malaysia.

Regional cooperation can benefit from utilising genetic evidence, particularly for migratory species like *A. sirm*. A fine example of how regional bodies co-operate in regional fishery management strategies is the ZoNéCo (Programme d’évaluation des ressources marines de la zone économique de Nouvelle-Calédonie) in New Caledonia that integrated genetic and complementary non-genetic data to manage the Spanish mackerel, *Scomberomorus commerson* stocks in Bélep, North Province of New Caledonia (von der Heyden et al., 2014). This project demonstrated that the area might host distinct reproductive stocks based on solid evidence of genetic structuring. Resultantly, a sound policy was introduced to ensure sustainable exploitation of the Spanish mackerel in New Caledonia. In another study, high genetic structuring attributed to limited ecological population connectivity of the shorefish, *Eleutheronema tetradactylus,* in four regions of northern Australia (Horne et al., 2011) recommended separate management of these four populations.

The presence of two discrete *A. sirm* stocks, the AS and the SCS, reinforces the need for regional cooperation among the maritime nations. This effort could be realized by referring to the management models applied in other regions through the facilitation of the Southeast Asian Fisheries Development Center (SEAFDEC). The genetic variability data could be used as an indicator to control the harvesting activities between participating countries, besides regulating the number of fish landing at a particular time and the fishing capacity of fishing vessels (Md Saleh et al., 2019).

Determining the population stock structure within the region (Malaysia with the neighboring waters) is also crucial for the sustainable fishery management of highly economically important species. This information could guide the fishery managers in determining potential fishing or spawning areas and planning a sustainable fishery policy strategy. For example, the population stock structure determination of spawning areas through DNA metabarcoding could be a way forward in sustaining broodstock management. In addition, the information could facilitate site recommendations for implementing closed areas during the critical life cycle, including spawning and nursery seasons, as suggested by Radhakrishnan et al. (2018) in their study on the Spanish mackerel, *Scomberomorus commerson*. The concept of genetic-based management is slowly gaining recognition in this region. For instance, SEAFDEC identifies potential closed areas under the “The South China Sea Fisheries Refugia Initiative” whereby genetic and non-genetic information is used to develop refugia of selected species such as the tiger prawn refugia in Sarawak, Malaysia (SEAFDEC, 2006). Despite that, there is an immense gap and challenges in incorporating genetic data for the development of fishery management strategies due to the lack of interest and knowledge among fishery managers (Benestan, 2019; von der Heyden et al., 2014). Furthermore, non-specialist may face difficulties understanding the genetic approaches in fishery management (Bowen et al., 2014), therefore, it is critical to address these challenges by maintaining and strengthening joint efforts between scientists and fishery managers. For instance, the integration of *A. sirm* genetic data along with life-history traits, tagging returns, parasitic loads, and microchemical variation of the hard skeletal structure in the fishery management policy (Ward, 2000; von der Heyden et al., 2014) would permit a holistic approach in sustainably managing *A. sirm* in this region. Such efforts will help achieve the Sustainable Development Goals (SDGs) specifically SDG14 to combat the global adverse effects of overfishing by 2030 (United Nations Sustainable Development, 2021) and effective conservation and sustainable fishery management.

## Conclusions

The mtDNA *Cyt b* marker revealed two highly differentiated *A. sirm* in the AS and the SCS and the neighboring waters. Demographic history and contemporary factors are identified as possible underlying reasons for the structuring observed. Meanwhile, cryptic diversity could explain the genetic disparity in the *A. sirm* population. Since two discrete stocks have been identified in this study, different management strategies would benefit the *A. sirm* population in the Southeast Asia.

## Supplemental Information

10.7717/peerj.13706/supp-1 Supplemental Information 1 Cytochrome sequences

## Additional Information and Declarations

### Data Availability

The following information was supplied regarding data availability: The sequences are available at GenBank: MZ040756 to MZ040877.
